# Oxidation-treated carbon nanotube yarns accelerate neurite outgrowth and induce axonal regeneration in peripheral nerve defect

**DOI:** 10.1038/s41598-023-48534-2

**Published:** 2023-12-09

**Authors:** Atsushi Kunisaki, Akira Kodama, Masakazu Ishikawa, Takahiro Ueda, Marcio D. Lima, Takeshi Kondo, Nobuo Adachi

**Affiliations:** 1 Department of Orthopaedic Surgery, Graduate School of Biomedical and Health Sciences, Hiroshima University, Hiroshima, Japan; 2 Nano-Science and Technology Center, LINTEC OF AMERICA, INC., Richardson, USA

**Keywords:** Neuroscience, Medical research, Neurology, Materials science

## Abstract

Carbon nanotubes (CNTs) have the potential to promote peripheral nerve regeneration, although with limited capacity and foreign body reaction. This study investigated whether CNTs hydrophilized by oxidation can improve peripheral nerve regeneration and reduce foreign body reactions and inflammation. Three different artificial nerve conduit models were created using CNTs treated with ozone (O group), strong acid (SA group), and untreated (P group). They were implanted into a rat sciatic nerve defect model and evaluated after 8 and 16 weeks. At 16 weeks, the SA group showed significant recovery in functional and electrophysiological evaluations compared with the others. At 8 weeks, histological examination revealed a significant increase in the density of regenerated neurofilament and decreased foreign body giant cells in the SA group compared with the others. Oxidation-treated CNTs improved biocompatibility, induced nerve regeneration, and inhibited foreign-body reactions.

## Introduction

Peripheral nerve injury is a relatively common cause of trauma^1^. Peripheral nerves have the ability to regenerate^2^, but is limited by long-term deficits or severe damage^3^. Graft surgery is required to repair nerve deficiency.

The gold standard for the repair of nerve defects is autologous nerve grafting, which leaves the internal nerve structure intact and promotes axonal regeneration^4^. However, it has disadvantages such as the need for surgery at other sites, loss of sensation and scarring at the donor site, and the possibility of neuroma formation due to the sacrifice of normal nerves^5^. Therefore, various artificial nerve conduits have been created and used as substitutes for autologous nerves. However, their use is limited to a gap of up to 3 cm and a diameter of up to 4 mm, and are mainly used for sensory nerves because they do not improve function of the motor nerves^6,7^. To overcome these limitations, new artificial nerve conduits need to be developed.

Here, we focused our research on carbon nanotubes (CNTs) as potential materials to promote peripheral nerve regeneration. CNTs are cylindrical nanostructures with diameter ranging from 0.4 to 40 nm and consisting of interwoven graphene sheets^8^. They have been studied as scaffolds for muscles, bones, and nerves because of their mechanical, electrical, thermal, and chemical properties and biocompatibility^9–11^. CNTs promote adhesion, diffusion, growth^12–15^, and electrical interactions in neurons, suggesting that they may stimulate neural circuits^16^. However, in vivo studies on the neuroregenerative effects of CNTs are still scarce^17,18^.

In our previous research, we confirmed that CNT fibers (cYarn^®^; LINTEC OF AMERICA INC., Richardson TX, USA) promote peripheral nerve regeneration^19^. In the study, we bridged a 15 mm rat sciatic nerve defect with cYarn^®^s and the regenerated axons crossing the CNTs; electromyographic findings and muscle weight ratio of the lower leg showed recovery of nerve function at 8 weeks, and the sciatic nerve functional index (SFI) showed improvement in gait function at 16 weeks. Histological evaluation confirmed that axons extended along the CNT fibers, and the percentage of axons extending beyond the implantation site increased significantly compared to the silicone tube group. These results confirmed the biocompatibility of CNTs in peripheral nerve regeneration.

Regrettably, its regeneration resulted in less than healthy nerve function, and the regenerated tissue had an accumulation of macrophages, causing a foreign body reaction. Therefore, we thought it necessary to improve the CNT to promote more effective regeneration.

In order to create better artificial nerve conduits, the materials must be biocompatible to minimize fibrosis, foreign body reactions, nerve shrinkage, and to prevent inflammation. They must also meet the technical requirements of good manufacturing processes, storage, and handling^20^. Factors that influence the biocompatibility of material surfaces include surface composition (type of functional groups and density/sign of charge), surface degradation, hydrophilic-hydrophobic character, wettability and surface free energy (SFE), topography (roughness and stiffness), and competitive protein binding^21^. Among these factors, wettability influences cell adhesion^22^, and is more enhanced on hydrophilic surfaces^23,24^. CNTs are hydrophobic, and this property may have worked against them in nerve regeneration.

CNTs have unique mechanical, electrical and physicochemical properties. Moreover, CNTs can be chemically modified to stimulate neurite growth. Chemical modification of CNTs can influence the length of neurite outgrowth, their number, branching, and the number of synaptic connections^25^. Therefore, CNTs modification is necessary to make them useful materials for nerve regeneration.

Previous research has shown that the oxidation of CNTs by methods such as ozone treatment modifies the carboxyl groups on the surface of CNTs^26^, which decreases the contact angle of water on CNTs and renders them hydrophilic^27^. Therefore, this study aimed to investigate whether CNTs made hydrophilic by oxidation treatment can further promote peripheral nerve regeneration and inhibit foreign body reactions and inflammation.

## Methods

### Production of the implants

Vertically aligned multiwall CNT (MWCNT) forests produced by chemical vapor deposition using acetylene as the carbon source and iron as the catalyst were used as the CNT source. The typical diameter and length of the MWCNTs were approximately 10 nm and 300 μm, respectively. The CNT yarn, named cYarn^®^ (LINTEC OF AMERICA INC.) was manufactured from the MWCNT forests using a dry drawing and spinning method^28–31^. The CNT yarns were ~ 15 μm in diameter (P-CNT).

**Figure 1:**
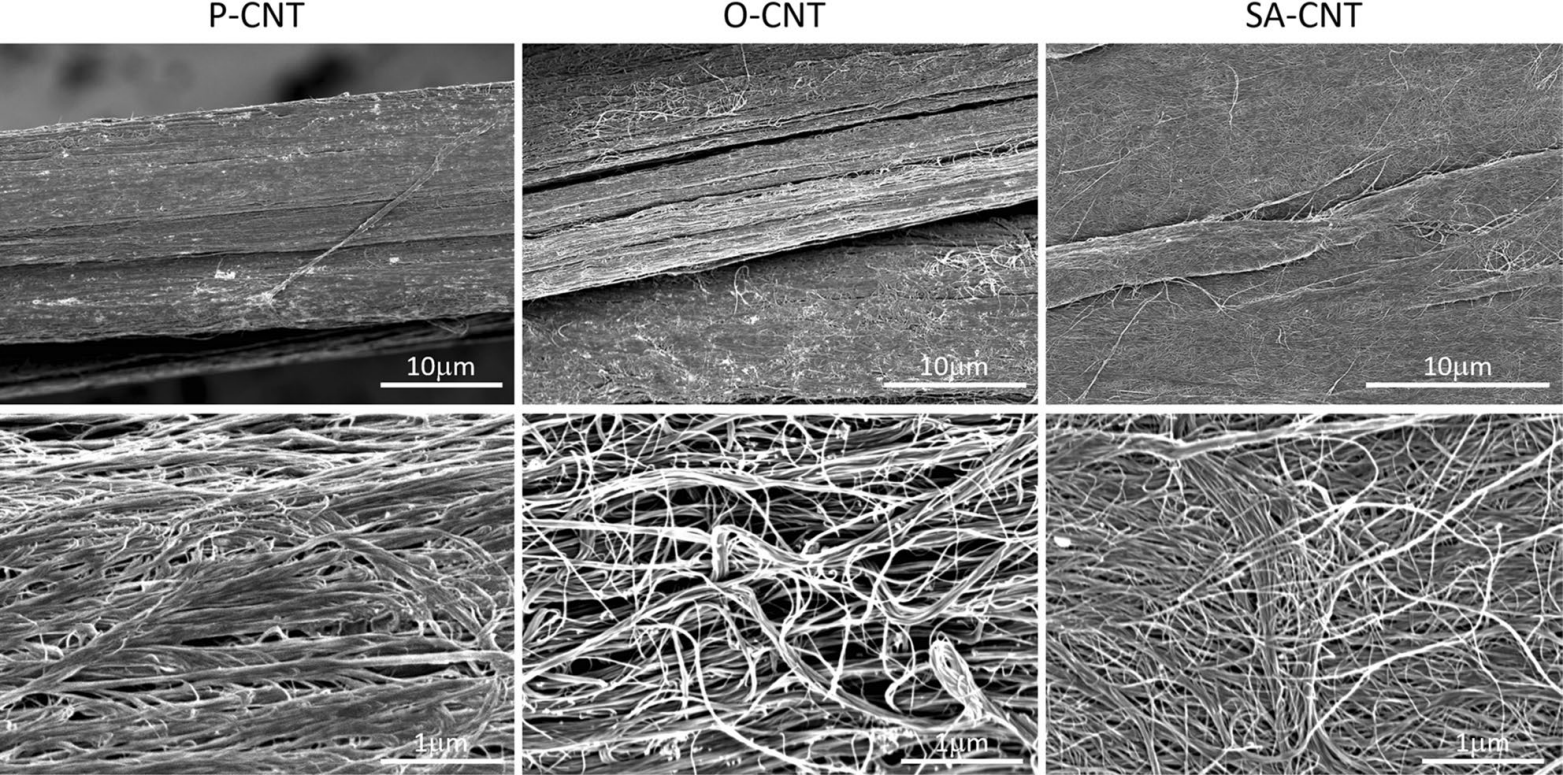
Morphology analysis using scanning electron microscopy of each carbon nanotube fibers.

Next, we treated the CNTs in ozone or with a strong acid to introduce oxygen functional groups. For ozonation, a mixture of 20% ozone and 80% atmospheric air was applied to the cYarn^®^ spools in a sealed chamber for 300 min (O-CNT) at room temperature and atmospheric pressure. Oxidized yarns were stored under ambient conditions at room temperature. For acid treatment, cYarn® bundles were treated with a mixture of two-thirds 70% HNO_3_ and one-third H_2_SO_4_ for 300 min and then rinsed with deionized water (SA-CNT). Scanning Electron Microscopy (SEM) images showed no significant changes in the fiber structure of cYarn due to oxidation treatment (Fig. 1).

### Evaluation of hydrophilicity of oxidized CNTs

**Figure 2:**
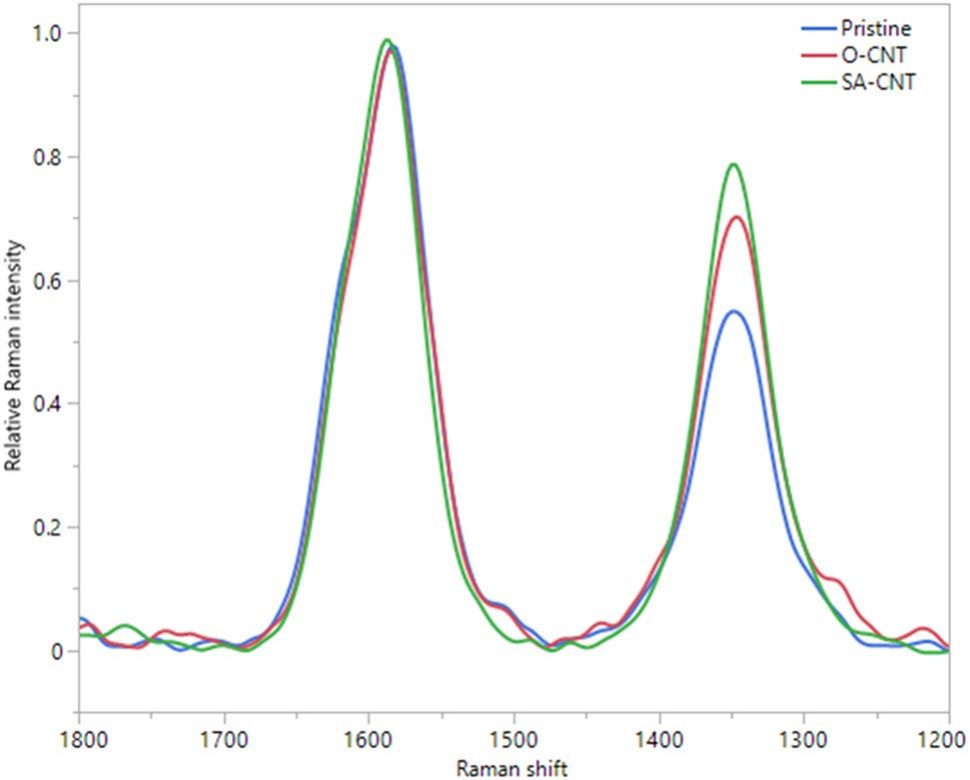
Raman spectra of the CNT groups.

Raman spectra of the cYarn^®^ samples were obtained to evaluate the crystallinity of CNT composed of cYarn^®^. Raman spectroscopy is a common method for characterizing structural changes in carbon materials and yields two prominent peaks centered at 1345 and 1585 cm^−1^ in the Raman spectrum of CNTs, assigned to defect-induced disorder (D) and graphite (G) modes, respectively (Fig. 2). The G/D ratio reflect amorphization, with decreasing values indicating an increase in defects^26^. With the oxidation treatment, the G/D ratio decreased in the order of O-CNT and SA-CNT, indicating the introduction of defects on the CNT (Table 1).

**Table 1:** G/D ratio for each CNT group.

Treatment type	G/D ratio
P-CNT	1.79
O-CNT	1.39
SA-CNT	1.28

A water wettability test was conducted to evaluate the hydrophilicity of cYarn^®^ with and without the oxidation treatments. First, we prepared yarn bundles with and without oxidation treatment as described in the sample preparation section. A total of 100 yarn bundles were inserted into silicone tubes (inner diameter: 1.5 mm; outer diameter: 2.5 mm), and the tubes with the yarn bundles were cut into approximately 2 cm pieces. The tubes were then placed in a water bath by touching one of the ends in the water to allow the yarn bundles to absorb water for three days. The weights of the tubes before and after the water absorption process were measured and the water absorption rate was calculated using the relative change equation. The water absorption rate increased in accordance with the G/D ratio, indicating an increase in the hydrophilicity of the yarn bundle (Table 2).

**Table 2:** Water absorption rate for each CNT group.

Treatment type	Water absorption ratio (%)
Control (no yarn)	0.0
P-CNT	1.5
O-CNT	4.3
SA-CNT	6.7

Regarding mechanical strength, a decrease in Young’s modulus and failure strength was observed for O-CNT. In the case of ozone treatment, measurements have been conducted for a 30 min treatment period, which demonstrated a reduction in Young’s modulus and a decrease in tensile strength. However, this evaluation has not been conducted for the 300 min treatment model in this study and is not presented as a quantified value.

In any case, the mechanical strength of CNT yarn is much stronger than that of any soft tissue, and changes in mechanical strength due to chemical treatment are unlikely to affect nerve regeneration.

For electrical resistivity, 250 bundles of cYarn^®^ were first treated with ozone and strong acid, and then silver paste was applied to both ends of the bundles and dried to form electrodes. The resistance and distance between these electrodes were then measured, and the resistivity per cm was calculated. It was confirmed that this increased with O-CNTs and decreased with SA-CNTs (Table 3). Leitsilber 200 Silver Paint was used for the silver paste and a Fluke 117 Digital Multimeter was used for the resistance measurements.

**Table 3:** Resistance for each CNT group.

Treatment type	Resistance (Ohm/cm)
P-CNT	8.0
O-CNT	9.6
SA-CNT	4.3

### Experimental animals and surgical procedures

Female Sprague Dawley rats (8 weeks old, weighing 200–230 g) were used in this study. Rats were housed in groups of three, fed a standard maintenance diet, and continuously provided with water.

A nerve conduit model was created by filling 17 mm long, 1.5 mm inner diameter silicon tubes with 15 mm CNT yarns of varying degrees of oxidation at a density of 5%. In order to analyze the performance of different oxidation of CNT fibers in vivo, we randomly sorted the animals into five groups of nine rats each:1) autograft (positive control group, AG, n = 9); 2) pristine CNT (P-CNT, n = 9); 3) ozonated CNT (O-CNT, n = 9); 4) strong acid treated CNT (SA-CNT, n = 9); and 5) silicon tube (negative control group, ST, n = 9).

For the surgery, anesthesia such as Rompun (20 mg/ml, Bayer Health Care, Leverkusen, Germany) and Ketalar (50 mg/ml, Daiichi-Sankyo, Tokyo, Japan) were injected intraperitoneally for each animal. A skin incision was made along the lateral femur, and the right sciatic nerve was exposed by dissecting the gluteus and biceps femoris muscles. The sciatic nerve was transected at the middle of the thigh using micro-scissors. For transplantation, 10 mm of sciatic nerve was removed. 1 mm segments of proximal and distal nerve ends were inserted into the conduits and attached with three sutures using an 8–0 monofilament nylon thread. In short, a 15 mm nerve gap was created. Before implantation, the conduits were soaked in NaCl after autoclave sterilization. For the autograft, a 15 mm long sciatic nerve was transected following a 180° rotation and reattached with 8–0 nylon.

All rats were euthanized with 100% carbon dioxide inhalation after an overdose of Rompun and Ketalar by intraperitoneal injection at the conclusion of the electrophysiological studies. We next harvested the sciatic nerves along with the conduits for further histological analysis. The silicon tubes were then removed. After post-fixation with 4% PFA overnight and immersion in a 20% sucrose solution for 48 h, the contents inside the silicon tube with proximal and distal nerve segments were embedded in OCT compound (Tissue-Tek, Sakura Finetek, Tokyo, Japan).

The nerve was sectioned sagittally or axially at an 8 µm thickness in accordance with a previously described method^32^. The sectioned surface was covered with an adhesive film (Cryofilm type IIC9, SECTION-LAB, Hiroshima, Japan), and frozen sections were prepared using a microtome (Cryostat HM520, Thermo Fisher Scientific K.K., Tokyo, Japan). The resulting sections were stained with hematoxylin and eosin (H&E) and each fluorescent stain, and immunohistochemical analysis was performed using a BZ-9000 microscope (Keyence Corp., Osaka, Japan).

### Sciatic functional index (SFI)

The SFI was calculated for every group to determine motor recovery every four weeks, until 16 weeks after the implantation. The test was conducted according to the method described by De Medinaceli et al.^33^. Animals were allowed to walk on a walking track, leaving footprints on white paper, and four parameters were measured from the animals’ footprints as follows: print length (PL), total spreading (TS), intermediate toes (IT) and distance to the opposite foot (TOF) on both uninvolved (normal) and involved (experimental) sides. The SFI formula was applied after measurement of each parameter.

### Electrophysiological study

The electromyography was evaluated at 8 weeks short-term and 16 weeks long-term. The examiner was blinded to the implants used for sciatic nerve reconstruction applied to each animal. The examinations were performed according to a previously described method^34^. The sciatic nerves proximal to the silicone tubes were exposed, and needle electrodes were placed in the gastrocnemius muscle. The nerves were stimulated with a constant current of 5.0 mA (0.2 ms square-wave pulses) using bipolar electrodes. The compound muscle action potentials (CMAPs) were recorded after stimulation using the Viking Quest system (Nicolet Biomedical, Madison, WI, USA). The onset latency and peak-to-peak amplitude of the CMAPs on experimental side were measured.

### Muscle weight ratio

The gastrocnemius and tibialis anterior muscles were weighed to calculate the ratio of the experimental side compared with the contralateral side.

### Immunohistochemistry

The sections were fixed in a 4% paraformaldehyde and methanol mixture for 30 s, washed with cold PBS three times for 3 min each time, and then blocked with 10% normal goat serum (Life Technologies, Carlsbad, CA, USA) at 4 °C for 1 h. Immunostaining was performed with anti-chicken NF protein (Abcam, Cambridge, UK) diluted 1:200 in PBS and anti-rabbit S100 protein (Abcam) diluted 1:200 in PBS. After incubation overnight at 4 °C, the sections were washed three times with PBS, then incubated with the secondary antibodies Alexa Fluor 488 goat anti-rabbit IgG and Alexa Fluor 568 anti-chicken IgG (Thermo Fisher Scientific K.K.), diluted in 1:200 in PBS for 1 h, and cover-slipped with DAPI counterstain for 5 min at room temperature.

Immunohistochemistry was performed using anti-mouse CD68 protein (Abcam), diluted 1:100 in PBS. After incubation overnight at 4 °C, the sections were washed three times with PBS, then incubated with the secondary antibody Alexa Fluor 350 anti-mouse IgG (Thermo Fisher Scientific K.K.), diluted in 1:200 in PBS for 1 h, and cover-slipped with DRAQ5 (Abcam) counterstain for 5 min at room temperature.

Photomicrographs of these sections were taken using a fluorescence microscope (Keyence, Osaka, Japan) connected to a digital camera and computer. The density of neurofilaments and Schwann cells was measured in two areas (proximal stamp:5 mm proximal to the proximal suture site; distal stamp:5 mm distal to the distal suture site) in randomly selected axial sections. The total number of DAPI-stained cells in the same square was counted. The number of macrophages were measured at one area (5 mm from the proximal stump) in randomly selected sagittal sections. ImageJ software (National Institutes of Health, Bethesda, MD, USA) was used for the analysis.

### Statistical analysis

Data are presented as mean ± standard error (SE). One-way ANOVA with post-hoc Bonferroni test was used to determine the differences between groups for CMAPs, muscle weight ratio, and immunohistochemical evaluation. Statistical significance was set at *p* < 0.05.

### Ethical approval and informed consent

Our research methods were reviewed and approved by the Ethics Committee of Hiroshima University (Hiroshima, Japan). All experiments were performed in accordance with relevant guidelines and regulations. This study was conducted in compliance with the ARRIVE guidelines.

## Results

### Electrophysiological evaluation

At eight weeks, CMAPs were detected in five of seven (71.4%) rats in the P-CNT group, six of eight (75.0%) rats in the O-CNT group, and nine of nine (100.0%) rats in the SA-CNT group. The mean latency and the mean amplitude respectively were 4.80 ± 0.40 ms and 451.5 ± 168 μV in P-CNT; 4.15 ± 0.19 ms and 702.7 ± 400.4 μV in O-CNT; and 3.97 ± 0.08 ms and 728.3 ± 157.9 μV in SA-CNT. No CMAPs were observed in the ST group. No significant differences were observed in the latencies and amplitudes between the P-CNT, O-CNT, and SA-CNT groups. At 16 weeks, CMAPs were detected in five of seven (71.4%) rats in the P-CNT group, five of five (100.0%) rats in the O-CNT group, and six of six (100.0%) rats in the SA-CNT group. Significant differences in latency were observed between the AG, O-CNT and SA-CNT groups compared with the P-CNT group. Significant differences in amplitude was observed between the AG group compared with the P-CNT group (Table 4).

**Table 4:** The compound muscle action potentials of the gastrocnemius for the five test groups at 8 weeks and 16 weeks after transplantation.

Group		AG	P-CNT	O-CNT	SA-CNT	ST
Recovery rate (%)	8 weeks	100 (8/8)	71.4 (5/7)	75 (6/8)	100 (9/9)	0 (0/9)
	16 weeks	100(5/5)	71.4 (5/7)	100 (5/5)	100 (6/6)	0 (0/5)
NCV (ms± SE)	8 weeks	4.61 ± 0.23	4.8 ± 0.4	4.15 ± 0.19	3.97 ± 0.08	–
	16 weeks	2.82 ± 0.28***	5.14 ± 0.3	3.28 ± 0.21***	4.01 ± 0.2*	–
Amplitude (µV± SE)	8 weeks	4791.1 ± 1518.2	451.1 ± 168	702.7 ± 400.4	728.3 ± 157.9	–
	16 weeks	17,149 ± 2019.3**	1841.2 ± 1038.3	5453.4 ± 2195.2	8215.5 ± 2813.5	–

NCV nerve conduction velocity, SE standard error. *p < 0.05, **p < 0.01, ***p< 0.001 (One-tailed ANOVA with Bonferroni compared with P-CNT).

### Sciatic functional index

**Figure 3:**
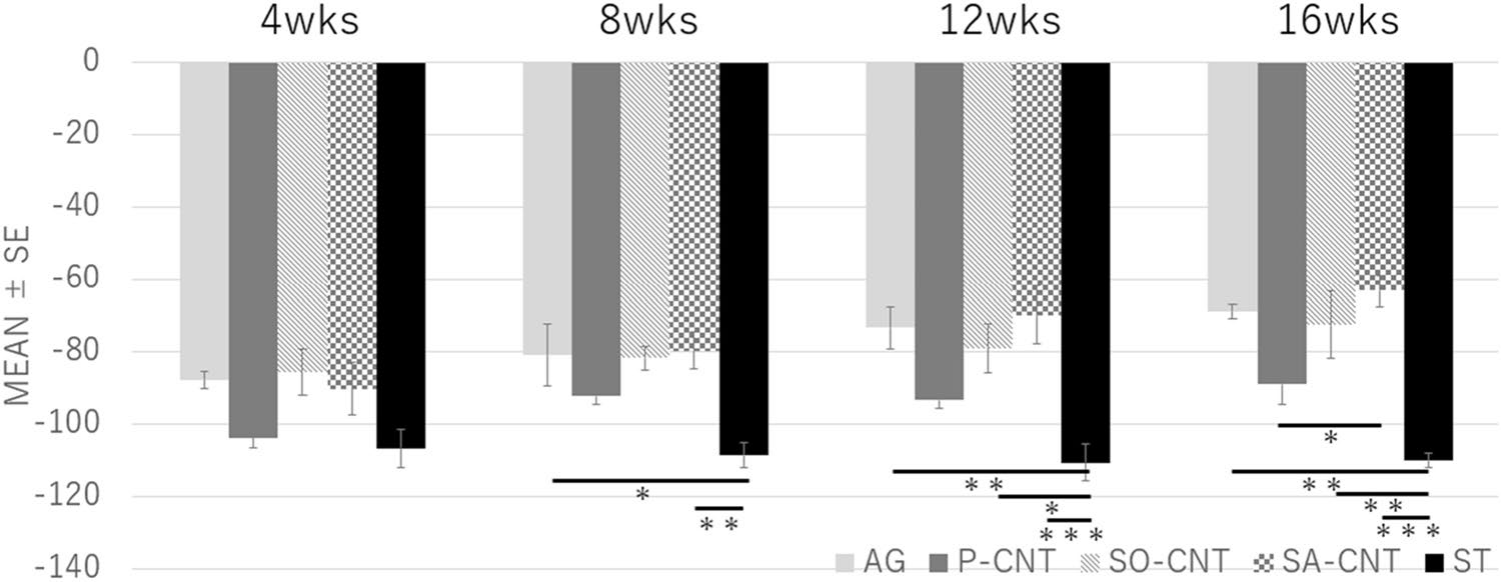
Result of SFI. *p < 0.05, **p < 0.01, ***p < 0.001 (One-tailed ANOVA with Bonferroni).

There was a trend of improvement in SFI as the oxidation strength increased. At 16 weeks, there was a significant difference in the SA-CNT group (− 63.2 ± 4.3) compared with the P-CNT group (− 88.9 ± 5.7) (Fig. 3).

### Muscle weight ratio

Muscle weight ratios are expressed as percentages of the contralateral side. At 8 weeks, the ratios of tibialis anterior and gastrocnemius were significantly greater in the AG group compared with the P-CNT group. At 16 weeks, the ratios were significantly greater in the AG group and SA-CNT group compared with the P-CNT group (Table 5).

**Table 5:** Lower limb muscle weight ratio for the five test groups at 8 and 16 weeks after transplantation.

Group		AG	P-CNT	O-CNT	SA-CNT	ST
TA (% ±SE)	8 weeks	50 ± 0.01***	25.4 ± 0.01	27.2 ± 0.01	28.9 ± 0.02	26.5 ± 0.01
	16 weeks	64.4 ± 0.01***	25 ± 0.03	25.6 ± 0.05	58.3 ± 0.05***	19.1 ± 0.02
GA (%± SE)	8 weeks	38.6 ± 0.02***	21.2 ± 0.00	20.9 ± 0.00	22.9 ± 0.01	18.7 ± 0.01
	16 weeks	68.1 ± 0.02***	22.6 ± 0.02	25.4 ± 0.05	48.8 ± 0.04***	16.9 ± 0.01

SE standard error. ***p < 0.001 (One-tailed ANOVA with Bonferroni compared with P-CNT).

### Microscopic findings

**Figure 4:**
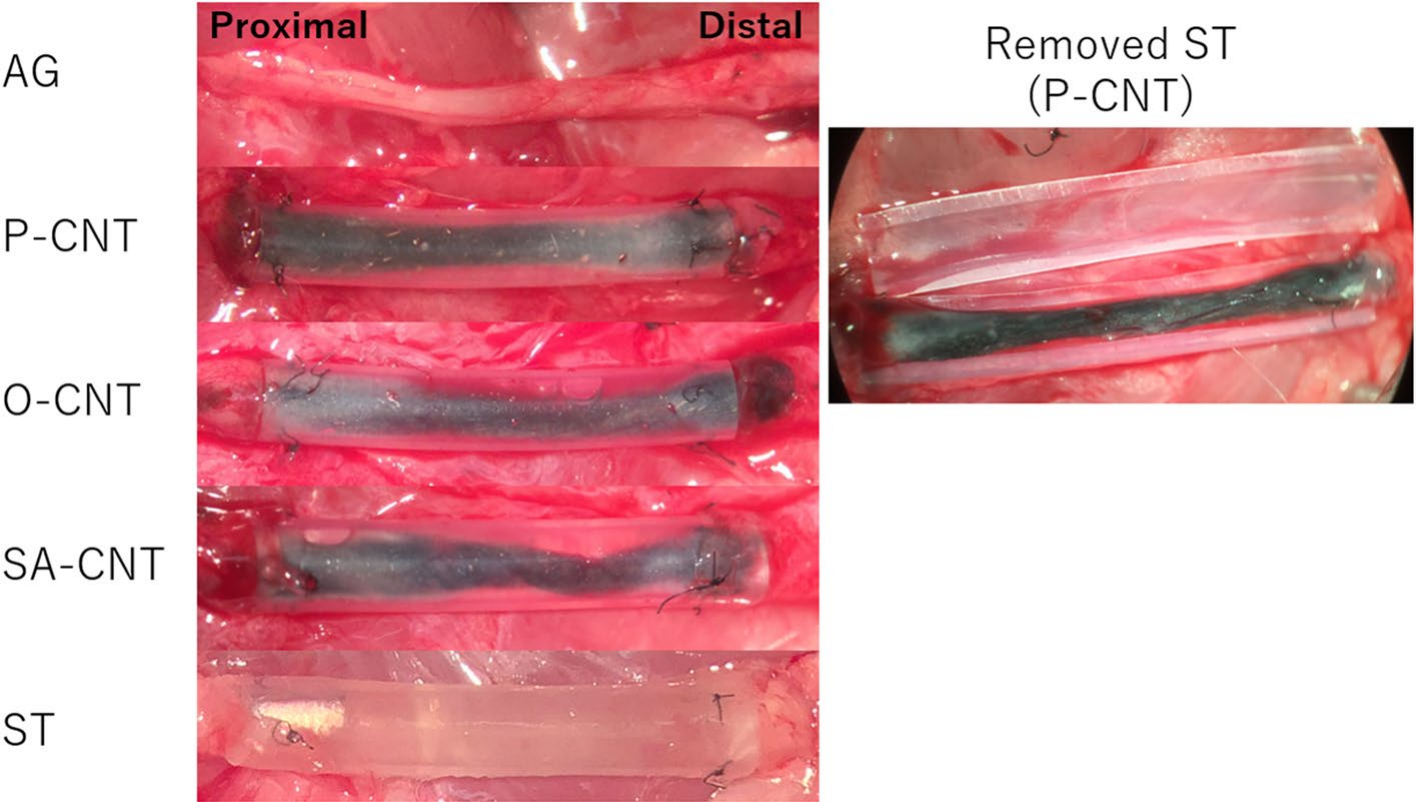
Intraoperative photograph showing the macroscopic appearance of the regenerated tissue and carbon nanotube yarn 8 weeks after implantation.

At 8 weeks postoperatively, white tissue and newly formed capillary vessels could be seen on the surface centered on the CNT cords in the CNT groups (P, O, and SA-CNTs). On the other hand, ST group could hardly nucleate cross-linked tissue, while AG group showed continuity as tissue (Fig. 4).

### Histological and immunohistochemical evaluation for axonal regeneration of excised sciatic nerve

**Figure 5:**
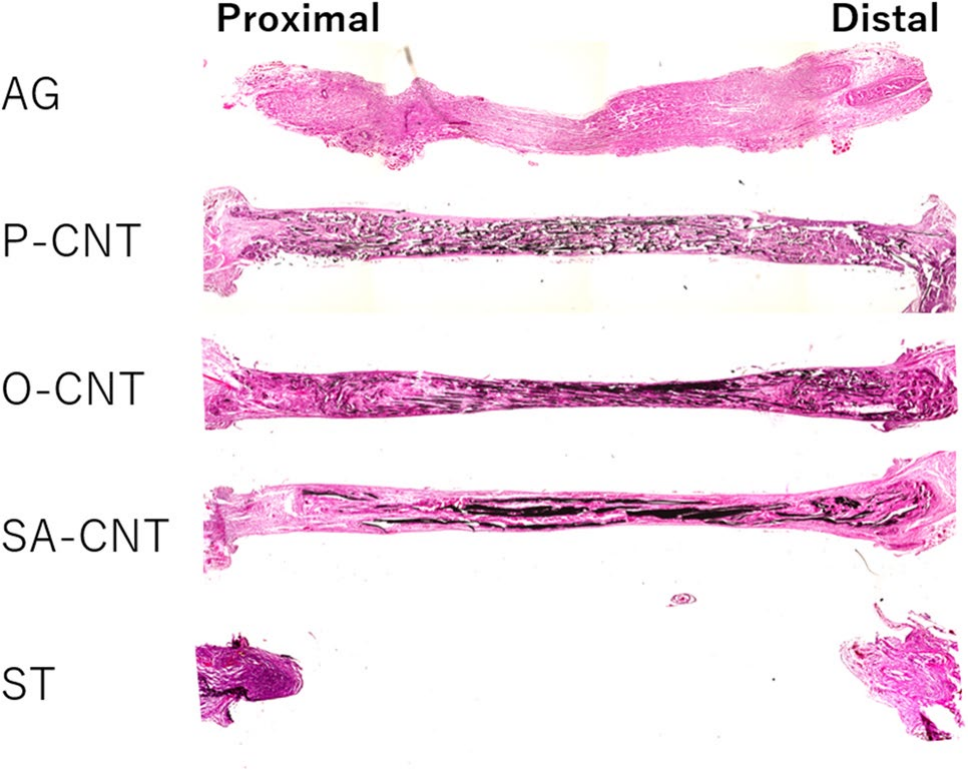
Representative light micrograph of a longitudinal section of a carbon nanotube nerve guide stained with hematoxylin and eosin 8 weeks after sciatic injury and repair.

**Figure 6:**
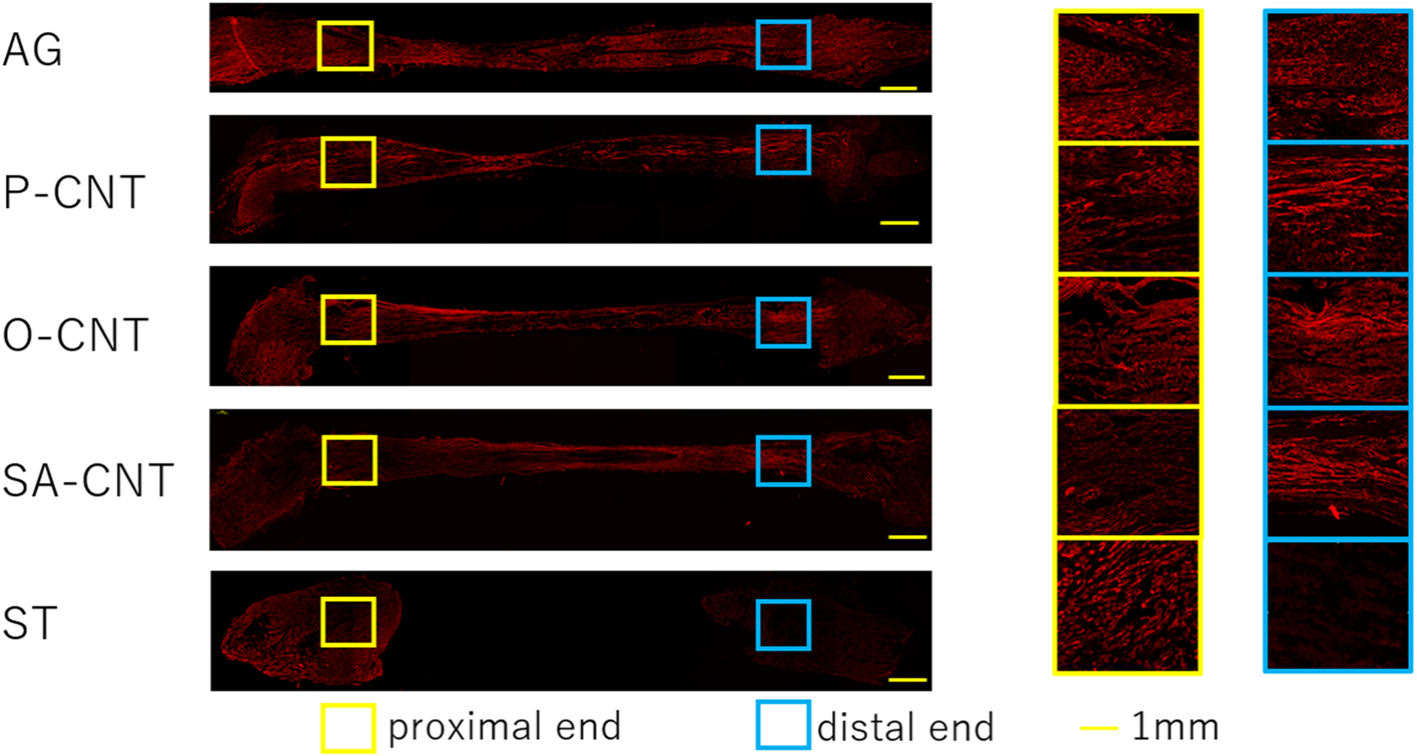
Longitudinal section of the regenerated tissue 8 weeks after the implantation. Sections were stained with neurofilament (axons, red). The figure on the right is an enlargement of the central and peripheral square areas in the artificial neural tube. Axonal extension to the periphery is observed in all but the ST group. Scale bar indicates 1 mm.

**Figure 7:**
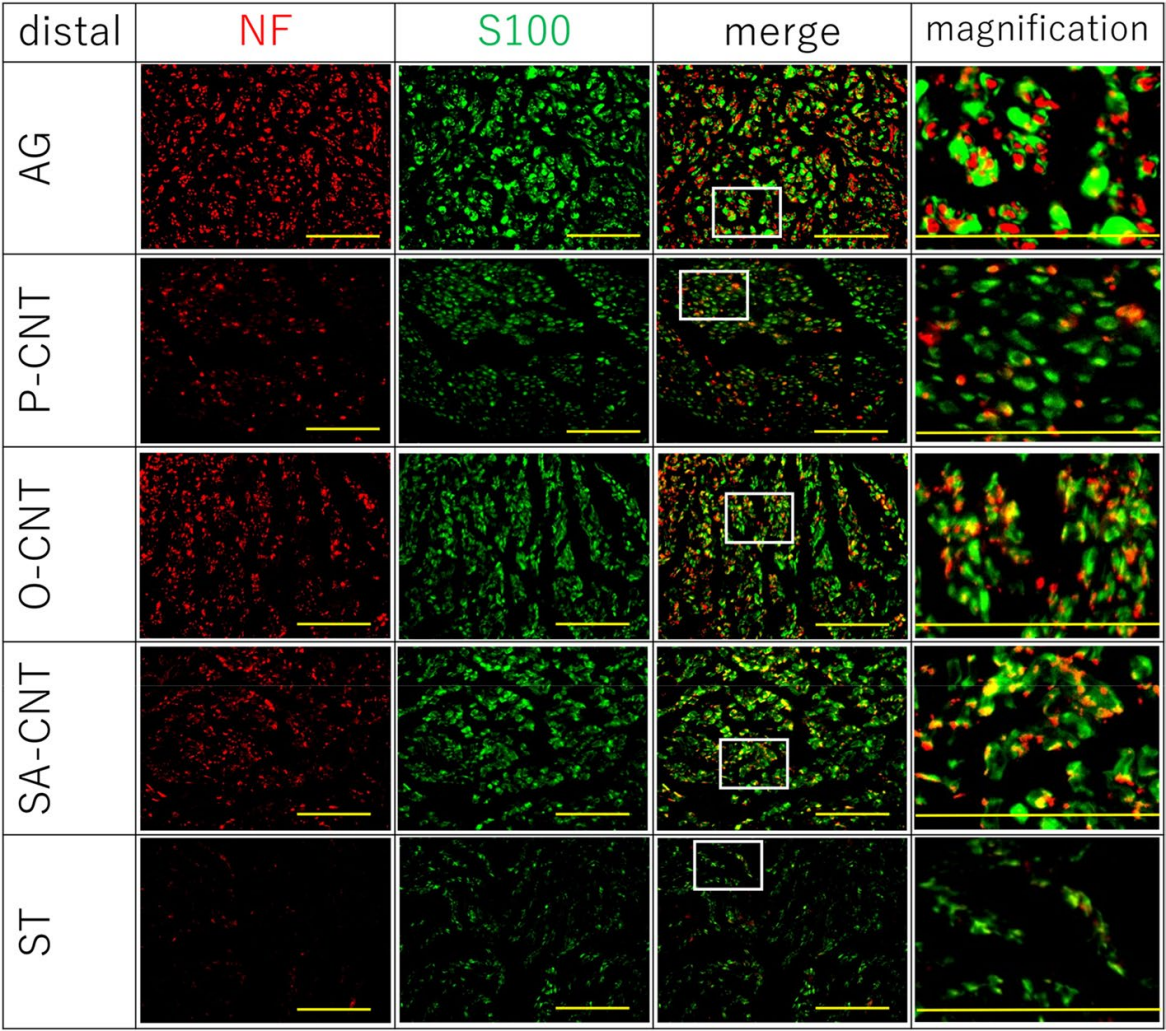
Transverse section of the distal nerve segments of each experimental group 8 weeks after the implantation. Sections were stained with neurofilament (axons, red), S100 (Schwann cells, green). The rightmost figure shows an enlarged square area. Regeneration of axons and Schwann cells is observed except in the ST group. Scale bar indicates 100 μm.

In longitudinal sections of regenerated tissue after 8 weeks, H&E staining confirmed that nerve defects were cross-linked in the regenerated tissue in all CNT groups (Fig. 5). Immunostaining of NFs showed that axons extended along the CNTs. In contrast, no cross-linked tissue or axonal regeneration was observed in the silicon group (Fig. 6). The percentage of regenerated axons and myelinated sheaths in the transverse section of the distal nerve end was expressed as the percentage of the proximal nerve end. All CNT groups showed an increase in the number of regenerating axons and myelin sheaths compared with the silicon group (Fig. 7). Furthermore, among the CNT groups, the SA-CNT group showed a significant increase in axonal regeneration compared with the P-CNT group. There was no statistically significant difference in the number of cell nuclei (DAPI) at the proximal and distal nerve ends between groups (Table 6).

**Table 6:** Immunohistochemical evaluation of axonal outgrowth and number of S100 positive cells in the proximal and the distal nerve segment in 8 weeks after the implantation.

		AG	P-CNT	O-CNT	SA-CNT	ST
Number of NF (± SE)	Proximal (no/mm^2^)	9741 ± 782	9334 ± 794	8622 ± 477	8914 ± 430	8419 ± 347
	Distal (no/mm^2^)	9016 ± 803	6847 ± 696	7475 ± 505	8269 ± 329	1692 ± 316***
	Distal/Proximal (%)	92.2 ± 3.6*	74.1 ± 5.6	86.6 ± 2.4	93.2 ± 1.5**	19.5 ± 3.0***
Number of S100 (± SE)	Proximal (no/mm^2^)	9694 ± 934	9584 ± 820	9122 ± 455	9508 ± 474	9144 ± 323
	Distal (no/mm^2^)	9463 ± 926	7297 ± 743	8092 ± 490	8886 ± 406	3176 ± 362***
	Distal/Proximal (%)	97.8 ± 3.0*	77.6 ± 6.7	88.9 ± 2.3	93.9 ± 1.9	34.7 ± 4.1***

NF neurofilament, SE standard error. *p < 0.05, **p < 0.01, ***p < 0.001 (One-tailed ANOVA with Bonferroni compared with P-CNT).

**Figure 8:**
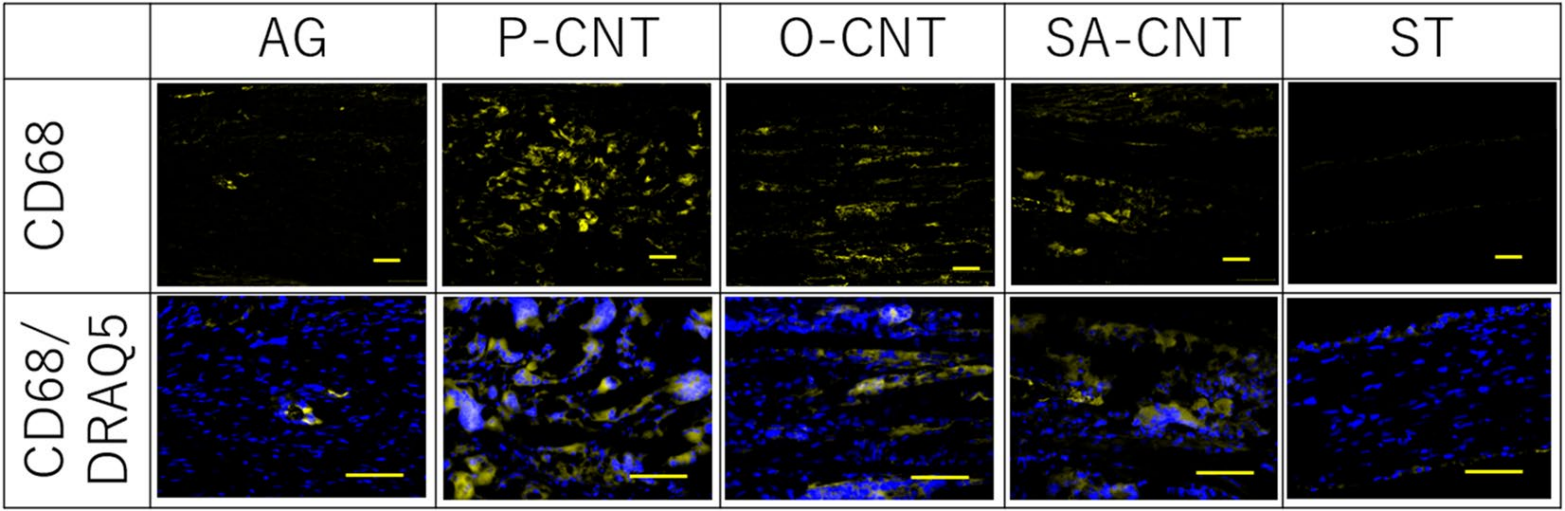
Longitudinal sectional area of 5 mm from the proximal stump 8 weeks after the implantation. Sections were stained with CD68 (FBGC, yellow), DRAQ5 (cell nuclei, blue). Scale bar indicates 100 μm.

CD68 staining in the proximal longitudinal section of regenerated tissue showed macrophages around the CNT fibers in each CNT group (Fig. 8). However, oxidative treatment decreased the number of macrophages, and a significant decrease was observed in the O-CNT (11.1 ± 0.7, *p* < 0.01) and SA-CNT (10.2 ± 0.8, *p* < 0.001) groups compared with the P-CNT group (15.4 ± 1, per 0.1 mm^2^ area) (Table 7).

**Table 7:** Immunohistochemical evaluation of the number of CD68-positive cells within the proximal transplanted nerve at 8 weeks after transplantation.

Group	AG	P-CNT	O-CNT	SA-CNT	ST
Macrophage (N/0.1 mm^2^ ± SE)	0.3 ± 0.1***	15.4 ± 1	11.1 ± 0.7**	10.2 ± 0.8***	–

SE standard error. **p < 0.01, ***p < 0.001 (one-sided ANOVA by Bonferroni compared to P-CNT).

**Figure 9:**
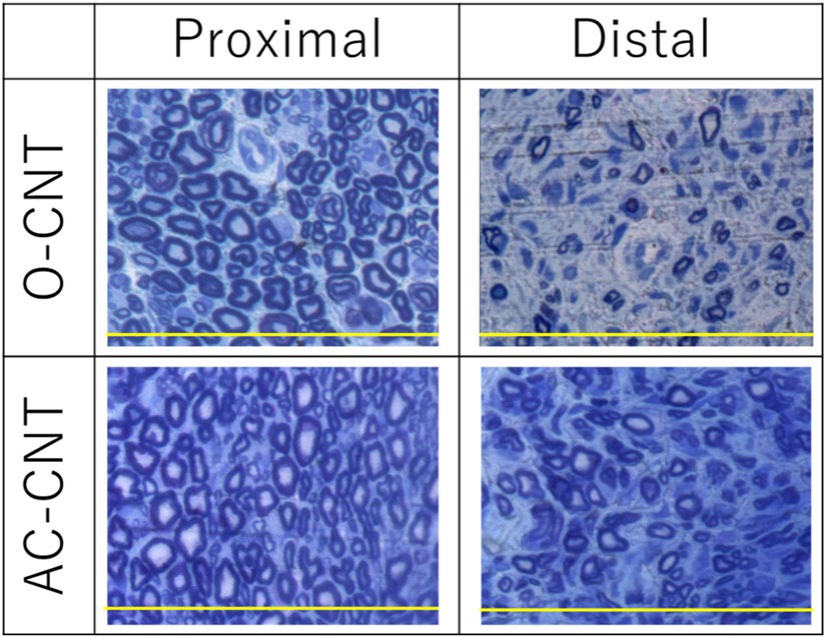
Transverse section of proximal and distal side 16 weeks after the implantation. Sections were stained with Toluidine blue. Scale bar indicates 100 μm.

Toluidine blue staining at week 16 showed that axon diameter and myelin sheath thickness in the SA-CNT group averaged 1.51 ± 0.06 μm and 0.88 ± 0.03 μm, respectively, 65.5% and 71.9% of the normal central side (Fig. 9). In the O-CNT group, it was 1.12 ± 0.05 and 0.74 ± 0.04, significantly increased in the SA group (*p* < 0.01, *p* < 0.05).

## Discussion

This study has illuminated that cYarn^®^, serving as a scaffold to facilitate nerve regeneration in 15 mm peripheral nerve defects, can be subject to chemical modification rendering it hydrophilic, thereby amplifying the promotion of nerve regeneration while concomitantly mitigating foreign body reactions. The outcomes of this investigation buttress the prospective clinical utilization of carbon nanotubes (CNTs) as a pre-fabricated material for artificial nerve conduits.

The surface properties of biomaterials that may modulate biological responses such as cell adhesion, shape, proliferation, and differentiation, include surface chemistry, topography, charge, wettability, and energy, and mechanical properties^35^. Previous study has indicated that CNT yarns have two major factors in their surface topography that favor nerve regeneration^19^. Firstly, CNT yarns are an aggregate of fibers of about 10 nm, closely resembling the dimensions of the cytoskeleton. These thin, twisted fibers possess a substantial surface area, which proves advantageous for both cell adhesion and axon elongation. Secondly, these fibers align longitudinally. This macrostructural alignment, where the fibers align parallel to the nerve run, is conductive to axon elongation. In this study, our focus was on surface wettability as a property capable of further enhancing the influence of CNTs on nerve regeneration. Surface wettability is understood to be hydrophilic and it has been reported that the more hydrophilic the surface is, the more enhanced is cell adhesion^36^. It has been demonstrated that fibronectin, a protein involved in cell adhesion, is adsorbed on more hydrophilic surfaces, while albumin, a protein that inhibits cell adhesion, is predominantly absorbed on hydrophobic surfaces^23,36^.

Furthermore, neural cells can exhibit a multifaceted array of morphologies that are acutely responsive to their local microenvironment. The manipulable chemical characteristics of CNTs can expose these cells to a diverse spectrum of stimuli^15,25^. For instance, unmodified CNTs demonstrate poor solubility and hinder cell adhesion. Nevertheless, the count of neurites, growth cones, and neurite branches can be modulated by adjusting the surface charge through chemical modification. Hu et al. chemically functionalized multi-walled nanotubes (MWNTs) with carboxyl groups, poly m-aminobenzene sulfonic acid (PABS), or ethylenediamine to procure CNTs possessing negative, zwitterionic, or positive surface charges, respectively. They also ascertained that positively charged MWNTs exhibited the most pronounced growth in rat hippocampal neurons, characterized by extensive branching and an elevated count of growth cones^37^. Furthermore, carbon nanotubes (CNTs) exhibit non-toxicity in in vitro environments^38^. Hydrophilic CNTs can modulate cell adhesion behavior, fostering cell adhesion, and influence neural patterning, thereby underscoring their biocompatibility^27,38^. This study demonstrated that the SA-CNT group, characterized by the highest hydrophilicity, exhibited significant axonal regeneration at 8 weeks after transplantation, as well as improved electrophysiology, muscle wet weight, and motor function at 16 weeks. This substantiated that higher hydrophilicity correlated with superior nerve regeneration. Regrettably, assessments of MARK/ERK and RAS activity, pivotal markers of Schwann cell differentiation and myelination, have not been conducted at an early stage. Nevertheless, within a protracted model, oxidized CNTs substantively augment the Schwann cell and axonal populace within the nerve beyond the transection site. The observed enhancement in myelination and axon elongation in the oxidized CNT group indirectly substantiates the amplification of Schwann cell induction and activity. Currently, the specific stimuli that promote the neuronal response remain obscure. However, the hydrophilization of CNTs through oxidation treatment, as demonstrated in this study, represents a methodology that can more effectively harness the unique physical, chemical, and biological properties of CNTs^25^ to instigate neural tissue regeneration.

On the other hand, a potential problem with biomaterials is that of foreign body reactions. These reactions can influence the biocompatibility (safety) of implanted biomaterials, significantly impacting short- and long-term tissue responses to the tissue engineering constructs employed in regenerative medicine. Foreign body reactions encompass processes such as protein adsorption onto biomaterials, adhesion of monocytes-macrophages, and the formation of foreign body giant cells (FBGCs) through macrophage fusion^39^. Hydrophilic surfaces have been reported to reduce cytokine expression by adherent inflammatory cells and inflammatory cells present in the surrounding exudate and inhibit macrophage fusion^40^.

The nature of adsorption on the biomaterial surface is also assumes a pivotal role in the foreign body response. Observations have indicated that vitronectin promotes the formation of FBGCs^41^, and that FBGC formation increases in plasma fibronectin knockout mice^42^. It is conceivable that the adsorption of fibronectin, induced by hydrophilicity, can modulate the foreign body response. Other reports suggest that hydrophilic surfaces contain a greater abundance of proteins associated with anti-inflammatory effects, whereas hydrophobic surfaces induce inflammatory responses through the adsorption of inflammatory cytokines and other inflammation-related proteins^43^. In another study, subcutaneous implantation of CNTs failed to induce cell necrosis as a cytotoxic sequela, while functionalized CNTs exhibited a propensity to mitigate inflammation when contrasted with their untreated CNT counterparts^44^. In our study, heightened hydrophilicity notably reduced macrophage accumulation (FBGCs) around cYarn^®^s. However, macrophage accumulation surpassed that observed in the autologous nerve group, and complete control over accumulation and fusion was not achieved. This could be attributed to the nanoscale diameter yet substantial longitudinal size of cYarn^®^s, which promotes macrophage accumulation and fusion (i.e., formation of FBGCs). On the other hand, signs of degradation and loss of structural integrity have been observed in functionalized CNTs, and there are reports of improved biodegradability^44^, suggesting that the CNTs used in this study may also become a more biocompatible material. Furthermore, silicon has been implicated in mediate macrophage fusion and apoptosis on biomaterial surfaces^45^, and the presence of silicon tubing might have influenced the foreign body reaction in our study. The use of a silicone tube as a support for external stability has been employed by other authors^46,47^, but as mentioned earlier, it can potentially amplify foreign body reactions. Consequently, it would be preferable to employ biodegradable materials for actual clinical applications.

In our study, we achieved hydrophilicity in CNTs through oxidation, thereby augmenting peripheral nerve regeneration and partially managing the foreign body response. Nevertheless, the SA-CNT group exhibited comparability with the autologous nerve graft group concerning SFI and histological regeneration indices but displayed inferiority in electrophysiological evaluation, muscle weight ratio, and foreign body reaction. However, CNTs can be subjected to various modifications, such as amination and growth factor binding^48,49^, which might offer even more precisely tailored approaches to nerve regeneration.

## Conclusion

This study provides evidence that carbon nanotubes (CNTs) act as scaffolds for peripheral nerve regeneration, and that hydrophilization of CNTs by oxidation enhances this regenerative effect. Our results demonstrate the potential of CNTs as valuable materials for nerve regeneration, whose chemical properties capable of being tailored for improved efficacy.

## Data Availability

We provide adequate assurances that they can comply with the publication's requirements for sharing materials. The datasets generated during and/or analyzed during the current study are available from the corresponding author on reasonable request.
